# Breaking Down Insulin Resistance

**DOI:** 10.1371/journal.pbio.1001483

**Published:** 2013-02-19

**Authors:** Caitlin Sedwick

**Affiliations:** Freelance Science Writer, San Diego, California, United States of America

**Figure pbio-1001483-g001:**
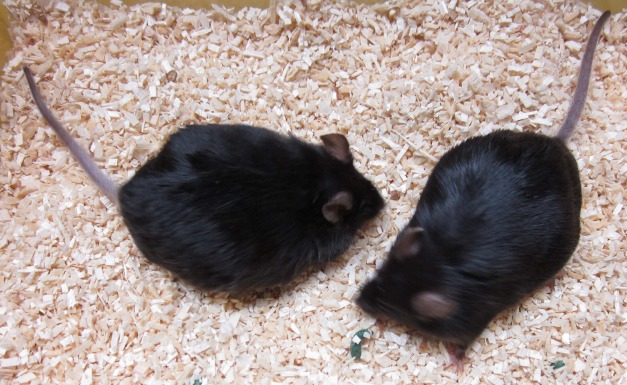
Both of these mice, which differ in their ability to mobilize fat stored in adipose tissues, become obese on a rich diet. The low fat-mobilizing mouse, however, is less prone to develop diabetes.

[Fig pbio-1001483-g001]The hormone insulin is critical for maintaining metabolic balance in the body. For example, skeletal muscle cells can't take up glucose on their own, and require insulin exposure to spur uptake and use of glucose. Meanwhile, the action of insulin promotes storage of fat in white adipose tissue (WAT), forcing skeletal muscle to rely on glucose for fuel, and also preventing liver cells from releasing glucose into the blood. Collectively, these effects ensure that blood glucose levels, which spike after a meal, aren't sustained at levels that can harm vulnerable tissues such as blood vessels.

But for reasons that remain unclear, insulin can sometimes lose its effectiveness so that cells become “insulin resistant” and stop taking up glucose despite the presence of insulin. These cells still need energy to live, so they start using fatty acids. WAT can generate fatty acid through lipolysis of its fatty acid stores to compensate. But this is risky for the body because high fatty acid levels can wreak havoc with metabolic functions and cause tissue inflammation. There's also evidence that excess fatty acid might actually drive insulin resistance by impairing signaling from the insulin receptor and blocking glucose transport. This may explain why insulin resistance is frequently observed in the obese, who have high WAT mass as a proportion of total body weight. However, high WAT mass is not always correlated with increased circulating fatty acid levels and insulin resistance, an observation that prompted an international collaboration headed up by Amandine Girousse and Dominique Langin to explore whether changes in WAT lipolysis rates drive insulin resistance in obese individuals.

The authors first examined lipolysis rates in WAT tissue samples taken from humans. Their analysis showed that people with elevated levels of WAT lipolysis are likely to also be insulin resistant, whereas people with low levels are unlikely to be insulin resistant. They also found that in people who had undergone stomach reduction surgery, those individuals with the biggest decrease in lipolysis rates also showed the biggest drops in insulin resistance. Therefore, lipolysis rates are highly correlated with insulin resistance in humans.

To get a closer look at the impact of WAT lipolysis on insulin resistance, the authors examined a mouse deficient in HSL, an enzyme that catalyzes lipolysis. Animals missing both genetic copies of HSL (HSL−/− mice) show drastic changes to their metabolism compared to wild-type mice, including an inability to become obese. This makes them unsuitable for the kinds of comparisons the researchers wanted to make, so they turned instead to animals hosting one normal and one defective copy of the gene encoding HSL (HSL+/− mice), whose WAT expresses half the normal amount of HSL.

Girousse et al. found that, compared to wild-type mice, HSL+/− mice fed a normal diet show decreased rates of WAT lipolysis. Interestingly, even though HSL+/− WAT releases less fatty acid, blood fatty acid levels in HSL+/− animals matched those found in wild-type mice. This is because other body cells reduce their fatty acid uptake so as to maintain the same circulating fatty acid levels as wild-type mice.

One possible outcome of lower lipolysis rates in HSL+/− mice is that fat stores would accumulate, making the mice more obese than wild-type mice when fed a high fat diet. But, the authors found, this is not the case. Moreover, HSL+/− animals of both normal weight and those that are obese retain better insulin sensitivity than do wild-type mice; HSL+/− mice show increased insulin-stimulated glucose uptake by skeletal muscle and WAT, and also improved insulin blockade of liver glucose release. The researchers explain that increased insulin sensitivity can be attributed to the fact that lower fatty acid secretion and uptake leads to improved insulin receptor signaling in HSL+/− mice. In addition, glucose transport mechanisms are improved, a conclusion supported by the group's experiments in cultured human adipocytes, which showed that knocking down HSL provokes increases in glucose uptake and expression of genes involved in glucose metabolism.

What happens to all the extra glucose taken up by these cells? The authors' data show that the sugar is being used as a fuel source in place of fats and also being incorporated into fatty acid, a process that may contribute to improvement of insulin sensitivity. Ultimately, lower levels of HSL induce a wholesale metabolic shift. Therefore, say Girousse et al., it might eventually be possible to reverse insulin resistance in people by targeting WAT lipolysis; indeed, they found that drug-mediated blockade of HSL activity allows wild-type mice to respond to insulin in the same way as HSL+/− mice. There are several ways WAT lipolysis could be inhibited, providing many drug candidates to develop and test.

**Girousse A, Tavernier G, Valle C, Moro C, Mejhert N, et al. (2013) Partial Inhibition of Adipose Tissue Lipolysis Improves Glucose Metabolism and Insulin Sensitivity without Alteration of Fat Mass. doi:10.1371/journal.pbio.1001485**

